# Straatsma Syndrome: A Case Series

**DOI:** 10.7759/cureus.29779

**Published:** 2022-09-30

**Authors:** Christina Karakosta, Konstantinos Paraskevopoulos, Anastasios Bisoukis, Konstantinos Bougioukas, Anna Kokolaki

**Affiliations:** 1 Ophthalmology, Korgialenio-Benakio Hellenic Red Cross Hospital, Athens, GRC; 2 Ophthalmolgy, Penteli General Hospital for Children, Athens, GRC; 3 Ophthalmology, Athens Public Eye Hospital, Athens, GRC; 4 School of Medicine, Health Sciences Departement, Aristotle University of Thessaloniki, Thessaloniki, GRC; 5 Ophthalmology, Penteli General Hospital for Children, Athens, GRC

**Keywords:** oct (optical coherence tomography), anisometropia, rnfl (retinal nerve fibre layer), anisometric amblyopia, high myopia

## Abstract

The aim of this article is to report two cases of Straatsma syndrome, a rare disease characterized by the traditional triad of unilateral myelinated retinal nerve fibres, axial myopia, and amblyopia.

The cases were a five-year-old and a three-year-old girl, both with unilateral myopia, visual acuity of 0.1 (decimal), and myelinated retinal nerve fibres in fundoscopy. Prescription of cycloplegic refraction findings and occlusion of the involved eye was attempted in both cases, but visual acuity did not improve. Poor-prognostic factors of Straatsma syndrome include a high degree of anisometropia and extensive myelination.

## Introduction

Straatsma syndrome is a rare disease described in 1979 for the first time by Straatsma [1]. It is characterized by the traditional triad of unilateral myelinated retinal nerve fibers (MRNF), amblyopia, and axial myopia [1]. Reverse Straatsma syndrome has also been reported and it is characterized by unilateral MRNF, amblyopia, and hyperopia [2]. Here, we report two cases of Straatsma syndrome.

## Case presentation

**Figure 1:**
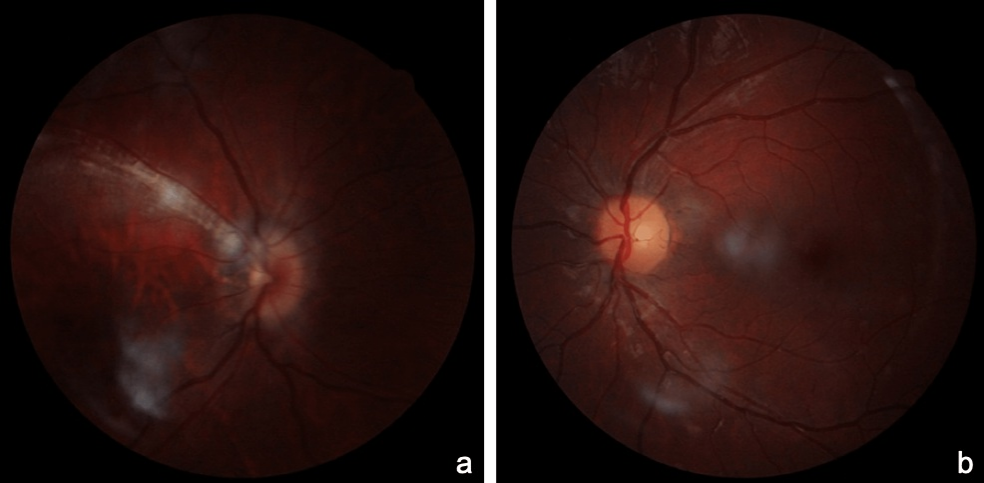
Fundus photography of the right eye showing myelinated retinal nerve fibers along the superior temporal arcade and fundus photography of the left eye within normal limits.

The first case was a five-year-old girl who presented at our department for a routine examination. Habitual visual acuity (decimal) was 0.1 (cc: −4.00 DS −3.50 DC × 175) in OD and 1.0 in OS sc. Cycloplegic refraction was −4.00 DS −4.00 DC × 180 in the right eye (OD) and Plano in the left eye (OS). The best corrected visual acuity (BCVA) was 0.1 in OD and 1.0 in OS. No strabismus was noted. Dilated fundoscopy of OD revealed a tilted disc and MRNF along the superior temporal arcade, without macular involvement (Figure 1a).

**Figure 2:**
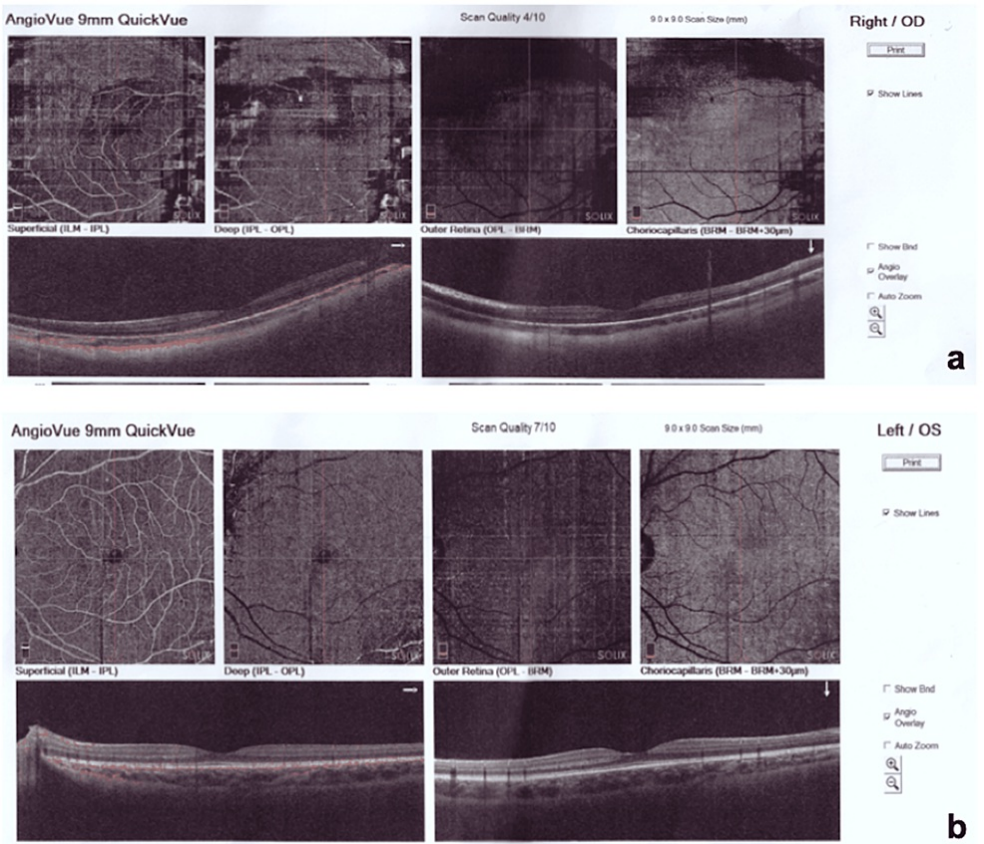
(a) OCT-angiography of the right eye within normal limits; (b) OCT-angiography of the left eye within normal limits. OCT: Optical coherence tomography.

Dilated fundoscopy was normal in OS (Figure 1b). Cycloplegic refraction was carried out along with intensive occlusion therapy. Contact lenses were used as well. However, BCVA did not improve in OD. Optical coherence tomography (OCT) and OCT-angiography were performed, which were within normal limits (Figure 2a-2b).

The girl was regularly examined in our department and 11 years later BCVA remained 0.1 in OD and 1.0 in OS.

**Figure 3:**
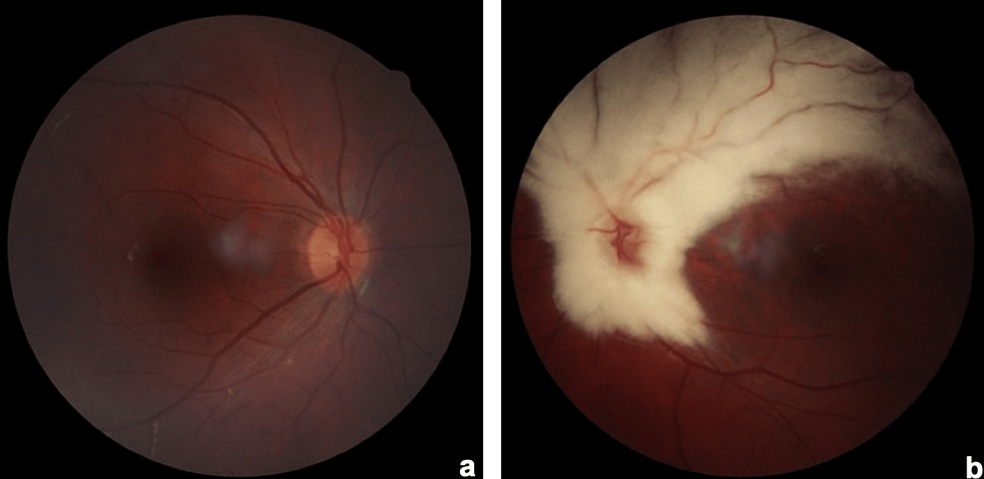
(a) Fundus photography of the right eye within normal limits; (b) fundus photography of the left eye showing extensive MRNF along the superior and inferior temporal arcade. MRNF: myelinated retinal nerve fibers.

The second case was a three-year-old girl. Cycloplegic refraction was plano in OD and −7.00 DS −1.50 DC × 180 in OS. BCVA was 1.0 in OD and 0.1 in OS. Dilated fundoscopy was normal in OD (Figure 3a), while in OS it revealed extensive MRNF around the peripapillary bundle and along the superior and inferior temporal arcades (Figure 3b).

**Figure 4:**
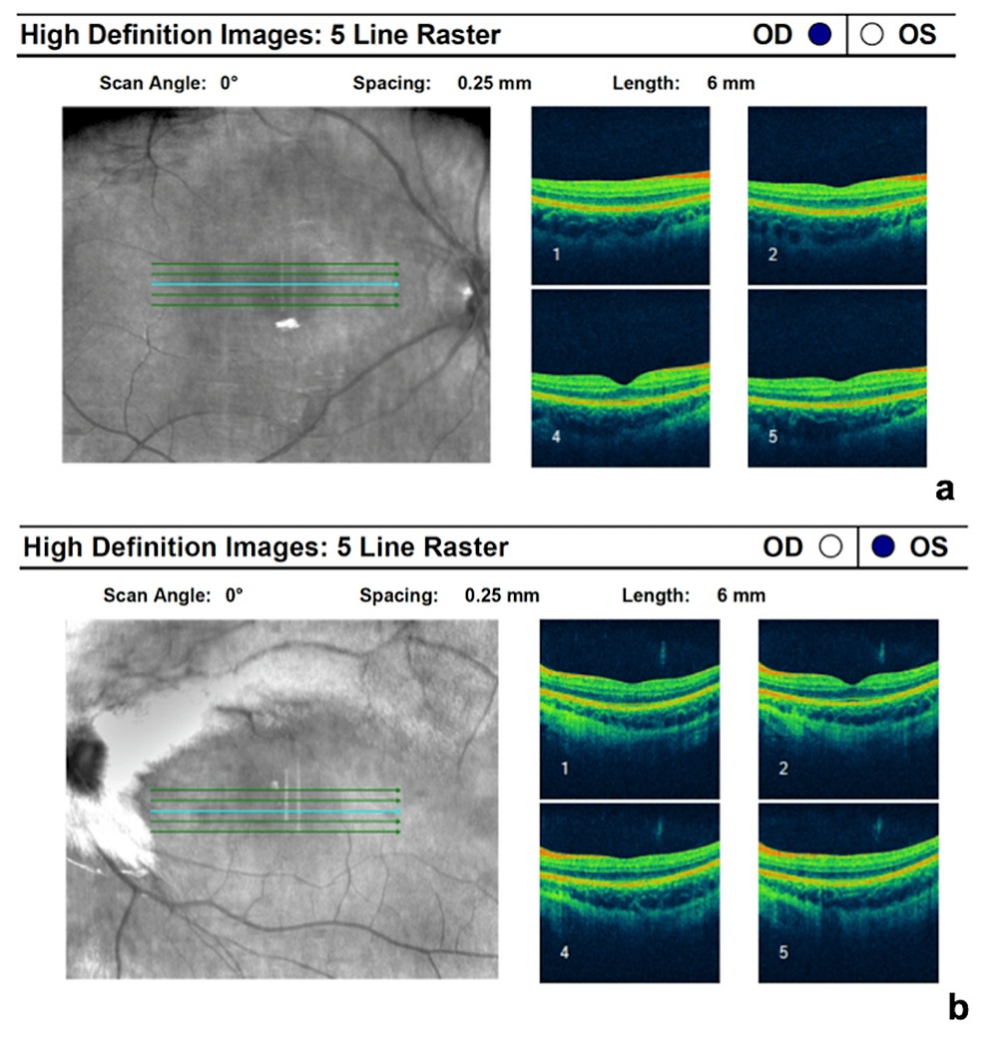
OCT scan of both eyes within normal limits. OCT: Optical coherence tomography.

OCT was normal in both eyes (Figure 4a-4b).

Intermittent exotropia of OS was present, but it improved after orthoptic exercises. Cycloplegic refraction findings and occlusion therapy were prescribed. The girl was regularly examined every three months in our department, and contact lenses with cycloplegic refraction were used as well. Nine years later, BCVA was measured at 1.0 in OD and 0.25 in OS.

## Discussion

Isolated MRNF is a rare clinical finding affecting 1% of the population [1]. A myelin sheath is normally present on retinal nerve fibers posteriorly to the lamina cribrosa. In contrast, MRNF abnormally appears with myelin sheath anteriorly to lamina cribrosa, as a white striated patch with feathered borders in the peripapillary area [3]. MRNF is usually a benign condition, but it may as well variably affect visual function based on the location and extension of myelination plaques and macular involvement [4]. In rare cases, it can be associated with other ophthalmological conditions, such as myopia and amblyopia, which is classified as Straatsma syndrome. Straatsma syndrome might also be associated with nystagmus [5], strabismus, hypoplasia of the optic disc, and heterochromia iridium [3,4,6]. In Straatsma syndrome, the prognosis of visual function is poor, despite occlusion therapy and full refractive correction, particularly in cases of a high degree of anisometropia, strabismus, extensive myelination, and macular involvement [3]. Thus, treatment of amblyopia may be challenging. The magnitude of anisometropia appears to be a critical prognostic factor. This means that a low degree of anisometropia is associated with better visual outcomes after the prescription of cycloplegic refraction findings and intensive occlusion therapy [4]. Strabismus in Straatsma syndrome is another factor associated with poor visual prognosis [4,7]. In both, our patient's initial BCVA was 0.1, with a high degree of anisometropia and similar spherical equivalents. However, BCVA of the affected eye improved in our second patient, despite more extensive MRNF and the presence of exotropia, probably due to the fact that occlusive therapy was started at an earlier age.

There are three types of MRNF, based on their location. In type 1, MRNF is present along the superior temporal arcade with continuity with the optic disc; in type 2, MRNF exists along both arcades with continuity with the optic disc; and in type 3, there is no continuity with the optic disc. Type 1 of MRNF is the most common, while type 2 has the worse prognosis for visual outcomes [3,8,9]. Our second patient presented with MRNF of type 2 and with a greater extension of myelination but had a better visual outcome than our first patient with MRNF of type 1.

Previous studies have proposed the presence of an organic etiology (abnormal foveal appearance on fundoscopy with a disruption of the ellipsoid zone on OCT) as a prognostic factor for poor response to occlusion therapy in patients with MRNF [3,8]. Both our patients had a normal foveal appearance and normal OCT scans.

## Conclusions

In conclusion, poor prognostic factors of Straatsma syndrome include a high degree of anisometropia, presence of strabismus, type 2 MRNF, macular involvement of MRNF, and occlusive therapy initiated at older ages. Extensive myelination is another poor prognostic factor, but even limited myelination may be associated with low visual acuity. In the presented cases, the extension of myelination did not seem to be such an important prognostic factor as the age at which occlusion therapy started. Even though Straatsma syndrome is associated with poor visual prognosis, intensive occlusion therapy should always be attempted, along with the prescription of a cycloplegic refraction, due to unexpected and variable responses to treatment.

## References

[1] Straatsma BR, Heckenlively JR, Foos RY, Shahinian JK (1979883). Myelinated retinal nerve fibers associated with ipsilateral myopia, amblyopia, and strabismus. Am J Ophthalmol.

[2] Shenoy R, Bialasiewicz AA, Al Barwani B (2011). Bilateral hypermetropia, myelinated retinal nerve fibers, and amblyopia. Middle East Afr J Ophthalmol.

[3] Sevik MO, Aykut A, Karaman NF, Şahin Ö (2021). Straatsma syndrome: should visual prognostic factors be taken into account? A case report. Turk J Ophthalmol.

[4] Tarabishy AB, Alexandrou TJ, Traboulsi EI (2007). Syndrome of myelinated retinal nerve fibers, myopia, and amblyopia: a review. Surv Ophthalmol.

[5] Juhn AT, Houston SK 3rd, Mehta S (2015). Bilateral Straatsma syndrome with nystagmus. Retin Cases Brief Rep.

[6] Vide-Escada A, Prior Filipe H (2017). Unusual Straatsma syndrome - how dogmatic is a bad prognosis?. Am J Ophthalmol Case Rep.

[7] Yalcın E, Balcı O, Akıngol Z (2013). Association of extensive myelinated nerve fibers and high degree myopia: case report. Indian J Ophthalmol.

[8] Ellis GS Jr, Frey T, Gouterman RZ (1987). Myelinated nerve fibers, axial myopia, and refractory amblyopia: an organic disease. J Pediatr Ophthalmol Strabismus.

[9] Straatsma BR, Foos RY, Heckenlively JR, Taylor GN (1981). Myelinated retinal nerve fibers. Am J Ophthalmol.

